# Clinical Implications of FADD Gene Amplification and Protein Overexpression in Taiwanese Oral Cavity Squamous Cell Carcinomas

**DOI:** 10.1371/journal.pone.0164870

**Published:** 2016-10-20

**Authors:** Huei-Tzu Chien, Sou-De Cheng, Wen-Yu Chuang, Chun-Ta Liao, Hung-Ming Wang, Shiang-Fu Huang

**Affiliations:** 1 Department of Public Health, Chang Gung University, Tao-Yuan, Taiwan, R.O.C; 2 Department of Anatomy, Chang Gung University, Tao-Yuan, Taiwan, R.O.C; 3 Department of Pathology, Chang Gung Memorial Hospital, Tao-Yuan, Taiwan, R.O.C; 4 Department of Otolaryngology, Head and Neck Surgery, Chang Gung Memorial, Tao-Yuan, Taiwan, R.O.C; 5 Division of Hematology/Oncology, Department of Internal Medicine, Chang Gung Memorial Hospital, Tao-Yuan, Taiwan, R.O.C; 6 Taipei CGMH Head and Neck Oncology Group, Tao-Yuan, Taiwan, R.O.C; Sudbury Regional Hospital, CANADA

## Abstract

Amplification of 11q13.3 is a frequent event in human cancers, including head and neck squamous cell carcinoma. This chromosome region contains several genes that are potentially cancer drivers, including *FADD* (Fas associated via death domain), an apoptotic effector that was previously identified as a novel oncogene in laryngeal/pharyngeal cancer. This study was designed to explore the role of FADD in oral squamous cell carcinomas (OSCCs) samples from Taiwanese patients, by assessing copy number variations (CNVs) and protein expression and the clinical implications of these factors in 339 male OSCCs. The intensity of FADD protein expression, as determined by immunohistochemistry, was strongly correlated with gene copy number amplification, as analyzed using a TaqMan CNV assay. Both FADD gene copy number amplification and high protein expression were significantly associated with lymph node metastasis (*P* < 0.001). Patients with both FADD copy number amplification and high protein expression had the shortest disease-free survival (DFS; *P* = 0.074 and *P* = 0.002) and overall survival (OS; *P* = 0.011 and *P* = 0.027). After adjusting for primary tumor status, tumor differentiation, lymph node metastasis and age at diagnosis, DFS was still significantly lower in patients with either copy number amplification or high protein expression (hazard ratio [H.R.] = 1.483; 95% confidence interval [C.I.], 1.044–2.106). In conclusion, our data reveal that FADD gene copy number and protein expression can be considered potential prognostic markers and are closely associated with lymph node metastasis in patients with OSCC in Taiwan.

## Introduction

Gene amplification refers to the somatically acquired increase in copy number of a restricted region of the genome, and this process is one of the underlying genomic mechanisms that results in overexpression of a dominantly acting oncogene [1]. Amplification is one of the distinct mechanisms that activate oncogenes. As we previously reported, amplification of oncogenes such as the epidermal growth factor receptor (EGFR) gene is accompanied by protein overexpression and can be associated with poor prognosis in human cancers [2]. Amplification at 11q13.3 is a common event in cancers from multiple anatomical sites, including head and neck squamous cell carcinoma (HNSCC) [3]. Several studies have demonstrated that there are at least three candidate oncogenes, *CCND1*, *FADD* (Fas associated via death domain) and *CTTN*, within *11q13*.*3* [3]. *CCND1* is a well-documented oncogene in breast, bladder, HNSCC, liver, and lung cancers [4–8]. *CTTN* also has well-established roles in the migration and invasion of tumor cells [9–10]. Its amplification has been reported in breast cancer, HNSCC, esophageal squamous cell carcinoma, hepatocellular cancer, melanoma and neuroblastoma. The third gene in this region, *FADD*, has been reported to be a critical apoptotic adaptor molecule: FADD interacts with cell surface death receptors and recruits caspases 8 and 10, thereby transmitting extracellular apoptotic signals to intracellular caspases and eventually resulting in apoptosis [11–13]. *FADD* has also been shown to enhance invasion in vitro, inhibit the necrosis of epithelial cells, and regulate the proliferation of epithelial and lymphoid cells [14–16]. *FADD* amplification has been demonstrated to play a role in laryngeal/pharyngeal cancer [17], and high protein expression (43%) was shown to be associated with worse survival in patients with tongue cancer [18].

In OSCC, we have shown that CCND1 is strongly associated with lymph node metastasis and patient survival [19]. The current study was designed to further investigate the role in OSCC of another important gene within *11q13*.*3*, FADD. The amplification and expression of *FADD* were evaluated in areca-quid (AQ)-associated OSCC and correlated with clinicopathological parameters.

## Materials and Methods

### Patients and specimens

This study was approved by the Chang Gung Medical Foundation Institutional Review Board (100-4358A3). The present study group consisted of 339 male patients diagnosed with primary OSCC who were admitted to Chang Gung Memorial Hospital, Lin-Kou, Taiwan, between 1999 and 2011. All cases were histologically confirmed and scored according to the recommendations for the reporting of specimens containing oral cavity, oropharynx and hypopharynx neoplasms by the Associations of Directors of Anatomical and Surgical Pathology (ADASP).[20] All patients signed informed consent for participation and were interviewed in a uniform manner before surgery by a well-trained interviewer. The questionnaire used in the interview sought detailed information on general demographics, as well as current and past cigarette smoking history, alcohol drinking and AQ chewing habits. Tumor tissue was collected, frozen immediately after excision in liquid nitrogen and stored at -80°C until DNA extraction. High-molecular weight DNA was purified as previously described [21]. All tumor specimens and tissue sections were retrieved from an archive.

### TaqMan CN assay using quantitative real-time polymerase chain reaction (qPCR) for *FADD*

FADD gene copy number was analyzed using pre-designed TaqMan copy number assay (Hs01625513_cn) (Applied Biosystems, Foster City, CA, USA) by qPCR on a 7500 Fast Real-Time PCR System (Applied Biosystems, Foster City, CA, USA) in our laboratory. The qPCR analysis was performed according to the MIQE guidelines [22]. The hydrolysis probe to determine the copy number of the FADD gene is located within exon 2. The reference probe targets a copy-number neutral region of the RNase P gene, serving as an internal standard. The quantitative duplex PCR assay was carried out in a 96-well optical plate with a total volume of 10 μl per well. The reactions included 2 μl of gDNA (~10 ng), 0.5 μl of *FADD* TaqMan Copy Number Assay solution (20×), 0.5 μl of *RNase P* TaqMan Copy Number Reference Assay solution (20×), and 5 μl of TaqMan Genotyping Master Mix (2×) with the final volume adjusted with sterile water. All reactions were performed in triplicate. Thermal cycling conditions included initial denaturation at 95°C for 10 mins, followed by 40 cycles of 15 s at 95°C and 60 s at 60°C. The number of copies of the *FADD* gene was determined by relative quantitation (RQ) using the comparative Cq (ΔΔCq) method, which requires a healthy control sample (diploid) as a calibrator in all amplifications. The RNase P gene was co-amplified with the FADD gene and served as an internal standard. FADD gene copy number status was defined by a comparative Cq (ΔΔCq) > 0.59 indicating amplification [23].

### Immunohistochemical analysis

**Fig 1:**
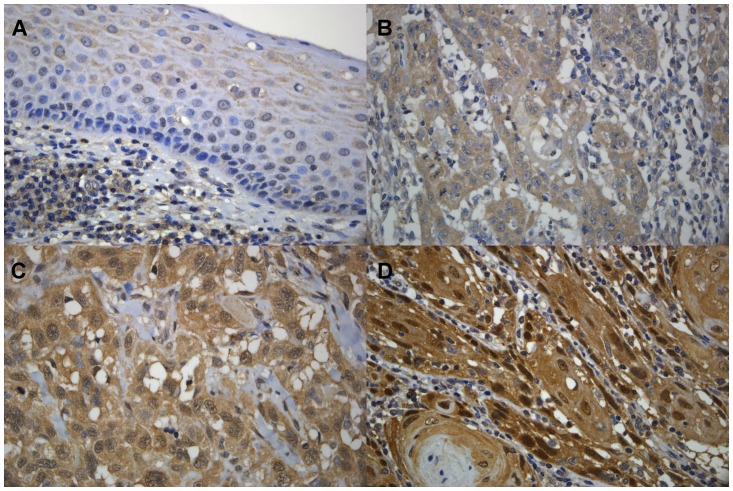
Immunohistochemical comparison of FADD expression. FADD protein expression in three OSCC patients and normal epithelium. (A), Normal epithelium showing weak cytoplasmic and nuclear staining as a reference. (B), Representative staining pattern of FADD 1+. (C), FADD 2+. (D) FADD 3+.

Paraffin-embedded tumor sections (1.5μm) were deparaffinized in xylene and absolute alcohol and retrieved with heat in 10 mM citrate buffer (pH 6.0). Immunostaining was performed using the UltraVision Quanto Detection System HRP (Thermo Scientific, Cheshire, UK) and a Lab Vision Autostainer 360 (Thermo Scientific, Cheshire, UK). In brief, slides were treated with hydrogen peroxide block reagent (Thermo Scientific) for 10 mins and rinsed with phosphate-buffered saline (PBS). After blocking non-specific binding with Ultra V Block reagent (UltraVision Quanto Detection System HRP kit; Thermo Scientific, Cheshire, UK), tissue sections were incubated with rabbit polyclonal anti-FADD antibody (1:500) (H-181; Santa Cruz Biotechnology, Santa Cruz, CA) for 1 hour at room temperature. The tissue sections were then washed with PBS and incubated with a secondary antibody from the UltraVision Quanto Detection System HRP at room temperature, and subsequently visualized by reaction with diaminobenzidine (DAB) used as the chromogen substrate (DAB Quanto kit; Thermo Scientific, Cheshire, UK). Finally, the slides were counterstained with hematoxylin and coverslipped with Permount and examined for the extent and intensity of cytoplasm in tumor cells and for background staining by the pathologist (WYC) in a blinded manner. In the present study, the normal epithelium present in most samples showed cytoplasmic staining of the suprabasal layer. In carcinoma cells, FADD protein expression was found mainly in the cytoplasm and distributed homogeneously in most tumors. In tumors with strong cytoplasm staining, there was usually some accompanying nuclear staining. The scoring criteria are as reported in Gibcus et al.[17]. In brief, using the normal epithelium as a reference for normal expression levels, we scored all samples according to the intensity as 0, 1+, 2+ and 3+. We categorized the FADD staining as low FADD expression when the intensity was 0 and 1+ (Fig 1A and 1B), and high FADD expression when intensity was 2+ and 3+ (Fig 1C and 1D).

### Statistics

Statistical analysis was carried out using the Statistical Package for the Social Sciences (SPSS) version 13. The correlations between FADD gene copy number or protein expression status and clinical parameters were examined by the χ^2^ test or Fisher’s exact test. Correlations between the FADD copy number and protein expression were tested with the χ^2^ test. Survival curves were constructed by the Kaplan-Meier method, and the curves were compared using the log-rank test. The Cox regression model was applied to simultaneously adjust all potential prognostic variables, including age, primary tumor status, lymph node status and differentiation. The results were considered significant if *P* < 0.05.

## Results

### Patient characteristics

Three hundred and thirty-nine patients with a diagnosis of OSCC were recruited into the study. The clinicopathological features of the patients are shown in Table 1. The most common primary sites were the bucca (44.8%, 152/339) and tongue (30.4%, 103/339). Overall, 85.6% (290/339) of the patients were cigarette smokers, 53.4% (181/339) were alcohol drinkers and 85.0% (288/339) chewed AQ. The primary treatment for these 339 patients was surgery; 232 (68.4%) patients underwent additional radiation therapy, and 89 (26.3%) underwent additional chemoradiotherapy. The median follow-up period was 60 months.

**Table 1:** Characteristics of the 339 OSCC patients.

Characteristics	
Age (years)	
Mean±SD	50.383± 11.164
Range	26–82
Site of primary tumor [No. of patients (%)]	
Bucca	155 (45.7)
Tongue	104 (30.7)
Other	80 (23.6)
Tumor stage [No. of patients (%)]	
Stage I	37 (10.9)
Stage II	59 (17.4)
Stage III	54 (15.9)
Stage IV	189 (55.8)
Differentiation [No. of patients (%)]	
Well differentiated	134 (39.5)
Moderately differentiated	182 (53.7)
Poorly differentiated	23 (6.8)
Cigarette smoking [No. of patients (%)]	
Yes	290 (85.6)
No	49 (14.5)
Alcohol drinking [No. of patients (%)]	
Yes	181 (53.4)
No	158 (46.6)
AQ chewing [No. of patients (%)]	
Yes	288 (85.0)
No	51 (15.0)

### FADD gene CNA and protein expression

*FADD* copy number amplification was found in 69 (20.4%) OSCC patients (Table 2). The levels of FADD protein expression were categorized as low and high expression subgroups according to the intensity of cytoplasmic staining (Table 2). We determined that 146 (43.1%) tumors had low expression, and 193 (58.7%) had high expression. High FADD protein expression was accompanied by increased FADD gene copy number. Sixty (60/69, 87.0%) patients with FADD gene amplification showed high FADD protein expression (S1 Table).

**Table 2:** Clinical association with FADD gene copy number and protein expression.

Characteristics	FADD gene copy number status			FADD protein expression status		
	Copy Neutral	Amplification	*P*-value	Low expression	High expression	*P-*value
	(N = 270)	(N = 69)		(N = 146)	(N = 193)	
Age						
< 50 yrs	140 (78.7)	38 (21.3)	0.633	67 (37.6)	111 (62.4)	**0.034**
≥ 50 yrs	130 (80.8)	31 (19.3)		79 (49.1)	82 (50.9)	
Subsites						
Bucca	127 (81.9)	28 (18.1)	0.602	75 (48.4)	80 (51.6)	0.186
Tongue	80 (76.9)	24 (23.1)		41 (39.4)	63 (60.6)	
Other	63 (78.8)	17 (21.3)		30 (37.5)	50 (62.5)	
Primary tumor status						
T1/T2	130 (80.3)	32 (19.8)	0.792	62 (32.3)	100 (61.7)	0.088
T3/T4	140 (79.1)	37 (20.9)		84 (47.5)	93 (52.5)	
Lymph node status						
LNM†-/ECS‡-	158 (86.8)	24 (13.2)	**0.001**	98 (53.9)	84 (46.2)	**< 0.001**
LNM+/ECS-	47 (77.1)	14 (23.0)	< 0.001*	19 (31.2)	42 (68.9)	< 0.001*
LNM+/ECS+	65 (67.7)	31 (32.3)		29 (30.2)	67 (69.8)	
Tumor differentiation						
Well	113 (84.3)	21 (15.7)	0.083	69 (51.5)	65 (48.5)	**0.011**
Moderate/Poor	157 (76.6)	48 (23.4)		77 (37.6)	128 (62.4)	
Skin invasion						
Yes	41 (89.1)	5 (10.9)	0.086	28 (60.9)	18 (39.1)	**0.009**
No	229 (78.2)	64 (21.8)		118 (40.3)	175 (59.7)	
Bone invasion						
Yes	73 (77.7)	21 (22.3)	0.574	43 (45.7)	51 (54.3)	0.538
No	197 (80.4)	48 (19.6)		103 (42.0)	142 (58.0)	
Perineural invasion						
Yes	71 (72.5)	27 (27.6)	**0.036**	31 (31.6)	67 (68.4)	**0.007**
No	199 (82.6)	42 (17.4)		115 (47.7)	126 (52.3)	
Vascular invasion						
Yes	8 (72.7)	3 (27.3)	0.473	3 (27.3)	8 (72.7)	0.363
No	262 (79.9)	66 (20.1)		143 (43.6)	185 (56.4)	
Lymphatic invasion						
Yes	34 (70.8)	14 (29.2)	0.102	19 (39.6)	29 (60.4)	0.599
No	236 (81.1)	55 (18.9)		127 (43.6)	164 (56.4)	
Invasion depth of tumor						
≥ 10 mm	163 (75.8)	52 (24.2)	**0.021**	91 (42.3)	124 (57.7)	0.716
< 10 mm	107 (86.3)	17 (13.7)		55 (44.4)	69 (55.7)	
Cigarette smoking						
Yes	230 (79.3)	60 (20.7)	0.709	120 (41.4)	170 (58.6)	0.127
No	40 (81.6)	9 (18.4)		26 (53.1)	23 (46.9)	
Alcohol drinking						
Yes	141 (77.9)	40 (22.1)	0.393	72 (39.8)	109 (60.2)	0.191
No	129 (81.7)	29 (18.4)		74 (46.8)	84 (53.2)	
AQ chewing						
Yes	233 (80.9)	55 (19.1)	0.172	125 (43.4)	163 (56.6)	0.767
No	37 (72.6)	14 (27.5)		21 (41.2)	30 (58.8)	

*χ^2^ trend test;

^†^LNM: lymph node metastasis;

^‡^ ECS: extracapsular spread

### Clinical implications of FADD gene CNA and protein expression

**Fig 2:**
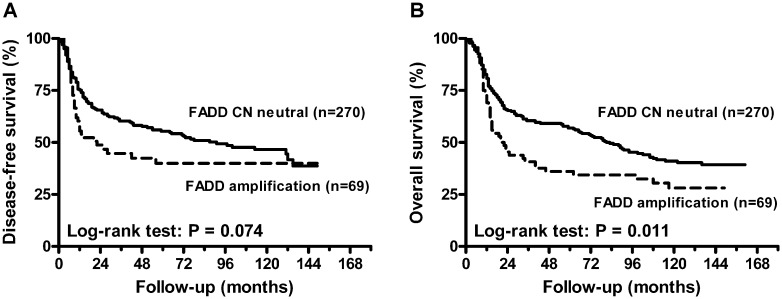
Survival curves based on analysis of the Fas-associated death domain (FADD) gene CNA. (A) Kaplan-Meier curves for disease-free survival (DFS).(B) Kaplan-Meier curves for overall survival (OS).

**Fig 3:**
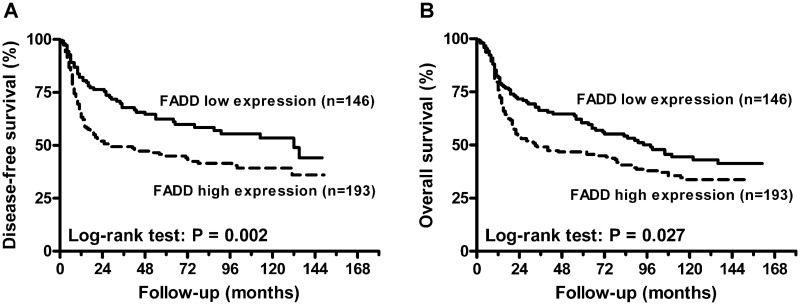
Survival curves based on analysis of Fas-associated death domain (FADD) protein expression. (A) Kaplan-Meier curves for disease-free survival (DFS).(B) Kaplan-Meier curves for overall survival (OS).

**Fig 4:**
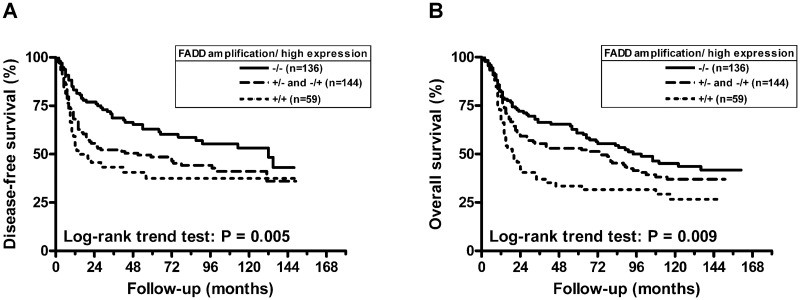
Survival curves based on combined Fas-associated death domain (FADD) gene CNA and protein expression analysis. (A) Kaplan-Meier curves for disease-free survival (DFS).(B) Kaplan-Meier curves for overall survival (OS).

As shown in Table 2, tumors with lymph node metastasis and lymph node extra-capsular spread (ECS) had a significantly higher frequency of *FADD* amplification (*P* ≤ 0.001). Similar results were observed for tumors with a high expression of FADD protein (*P* < 0.001). Furthermore, high FADD protein expression was associated with younger age at diagnosis (*P* = 0.034) and a higher grade of tumor differentiation (*P* = 0.011). We further analyzed the relationship between FADD and patient prognosis by univariate analysis (Fig 2). The results showed that FADD gene amplification was associated with poor overall survival (OS) (Fig 2B, *P* = 0.011) [hazard ratio (HR) = 1.527, 95% confidence interval (CI), 1.098–2.123] (Table 3). High FADD protein expression was associated with poor disease-free survival (DFS) (Fig 3A) (HR = 1.684, 95% CI, 1.209–2.345) and OS (Fig 3B) (HR = 1.387, 95% CI, 1.035–1.859). The patients with both FADD gene amplification and protein overexpression showed worse DFS (Fig 4A, *P* = 0.005) and OS (Fig 4B, *P* = 0.009). In addition to FADD, primary tumor status (HR = 1.676, 95% CI, 1.256–2.238) and lymph node metastasis (HR = 1.931, 95% CI, 1.310–2.846) were significantly associated with a poorer OS. Lymph node ECS was also significantly associated with a poorer DFS (HR = 3.124, 95% CI, 2.202–4.432). After adjusting for age at diagnosis, primary tumor status, lymph node status and tumor differentiation by multivariate Cox regression, patients with FADD gene amplification or high protein expression had the worst DFS (*P* = 0.028, HR = 1.483, 95% CI, 1.044–2.106) (Table 4). Of the FADD gene copy neutral cases, we observed that 49.3% (133/270) of cases displayed a high expression level of FADD protein independently of gene copy number alteration (S1 Table). High FADD protein expression was also associated with young age at diagnosis (*P* = 0.029) and lymph node metastasis (*P* = 0.025) in this subgroup (S2 Table). High FADD protein expression also was associated with DFS in this subgroup (HR = 1.690, 95% CI, 1.173–2.435) (S3 Table). After adjusting for age at diagnosis, primary tumor status, lymph node status and tumor differentiation using multivariate analysis, patients with high FADD protein expression also showed a poorer DFS (HR = 1.575, 95% CI, 1.078–2.300) (S4 Table).

**Table 3:** Univariate Cox regression model of prognostic covariates in 339 patients with OSCC: disease-free and overall survival.

Characteristics	DFS	*P*-value	OS	*P*-value
	HR (95% CI)		HR (95% CI)	
Age				
< 50 yrs	1		1	
≥ 50 yrs	0.914 (0.667–1.252)	0.575	1.119 (0.843–1.487)	0.437
Primary tumor status				
T1/T2	1		1	
T3/T4	1.145 (0.837–1.567)	0.398	1.676 (1.256–2.238)	**< 0.001**
Lymph node status				
LNM†-/ECS‡-	1		1	
LNM+/ECS-	1.548 (0.994–2.410)	0.053	1.931 (1.310–2.846)	**< 0.001**
LNM+/ECS+	3.124 (2.202–4.432)	**< 0.001**	3.018 (2.191–4.156)	**< 0.001**
Tumor differentiation				
Well	1		1	
Moderate/Poor	1.194 (0.865–1.646)	0.281	1.344 (1.000–1.807)	**0.050**
FADD CN status				
Copy neutral	1		1	
Amplification	1.393 (0.963–2.016)	0.079	1.527 (1.098–2.123)	**0.012**
FADD expression				
Low expression	1		1	
High expression	1.684 (1.209–2.345)	**0.002**	1.387 (1.035–1.859)	**0.029**

^†^LNM: lymph node metastasis;

^‡^ ECS: extracapsular spread;

CN: copy number

**Table 4:** Multivariate Cox regression model of prognostic covariates in 339 patients with OSCC: disease-free and overall survival.

Characteristics	DFS	*P*-value	OS	*P*-value
	HR (95% CI)		HR (95% CI)	
Age				
< 50 yrs	1		1	
≥ 50 yrs	1.058 (0.766–1.462)	0.732	1.231 (0.922–1.645)	0.159
Primary tumor status				
T1/T2	1		1	
T3/T4	0.990 (0.716–1.368)	0.950	1.469 (1.092–1.976)	**0.011**
Lymph node status				
LNM†-/ECS‡-	1		1	
LNM+/ECS-	1.448 (0.920–2.280)	0.110	1.871 (1.258–2.783)	**0.002**
LNM+/ECS+	3.003 (2.067–4.363)	**< 0.001**	2.702 (1.923–3.797)	**< 0.001**
Tumor differentiation				
Well	1		1	
Moderate/Poor	0.927 (0.665–1.292)	0.653	1.042 (0.768–1.415)	0.790
FADD status				
FADD CN-/low expression	1		1	
FADD CN+ or high expression	1.483 (1.044–2.106)	**0.028**	1.270 (0.933–1.729)	0.128

^†^LNM: lymph node metastasis;

^‡^ ECS: extracapsular spread;

CN: copy number

## Discussion

Gene amplification is a well-known mechanism that results in an increase in the expression of genes involved in oncogenesis, tumor development, or multidrug resistance [24]. Amplification of chromosome 11q13 is frequently found in human cancers and is prominent in HNSCC (30–62% of cases) [3]. *CCND1* and *CTTN* have usually been considered to be the driver genes in the 11q13.3 locus. *FADD*, which is also located within 11q13.3, was previously identified as a novel cancer gene in laryngeal/pharyngeal cancer [17].

In the current study of male HNSCC patients, we showed that *FADD* amplification was present in 69 of 339 cases (20.4%), and gene amplification was associated with a higher incidence of lymph node metastasis (LNM) and extracapsular spread (ECS). The frequency of amplification is lower than in a previous report on tongue SSC, in which tumor tissue was hand-dissected from 30 samples and transcripts were amplified by real-time PCR (43.3%, 13/30) [18]. To our knowledge, the present study is the largest cohort of male patients with OSCC in which the role of FADD gene copy number has been assessed in the tongue, bucca and other locations of the oral cavity. Although the association between *FADD* amplification and LNM has not been observed in previous reports, the strong positive relationship between 11q13 amplification and lymph node status has been reported in HNSCC [25].

In most samples, *FADD* amplification was accompanied by high FADD protein expression (87.0%, 60/69), as expected. However, the proportion of samples with high FADD expression (56.9%) was much higher than that with FADD gene amplification (20.4%). This discrepancy between gene alteration and protein expression suggests that FADD can be overexpressed by other mechanisms such as post-transcriptional modification or altered protein expression from the interactions of other related genes in the absence of DNA amplification, as has been observed for the MDM2 gene in the 12q13-15 amplicon in human sarcoma [26]. In addition to lymph node metastasis, high expression of FADD was significantly associated with young age at diagnosis and poorer tumor differentiation. There were no difference in the distribution of primary tumor stage, nodal metastasis between young and old age groups. In the environmental carcinogen exposures, the frequency of alcohol drinking (*P* = 0.017) and areca quid chewing (*P* < 0.001) were significantly higher in the young age group whilst the cigarette smoking was not (*P* = 0.249). Further analyzing the relationships between cigarette, areca quid, alcohol consumption and FADD overexpression, no significant associations exist between them. The underlying mechanisms that cause the higher frequency of FADD overexpression in young age group need further investigation. However, the effect of FADD gene amplification and overexpression on survival was not influenced by age because we adjusted the variable in the multivariate analysis. The relationship between FADD expression and tumor differentiation was similar to that in early stage tongue cancer [18]. Recently, studies have demonstrated that FADD plays a crucial role in cell growth by interacting with the adenylate kinase 2 (AK2)/dual-specificity phosphatase 26 (DUSP26) protein complex and could be associated with tumor cell differentiation [27].

Similarly to FADD amplification, high FADD protein expression is also significantly associated with lymph node metastasis (*P* < 0.001). To evaluate the exact effect of FADD protein expression on lymph node metastasis, we analyzed the relationship between high FADD protein expression and lymph node status in the *FADD* copy neutral subgroup. Notably, the association between high FADD protein expression and lymph node status was retained (*P* = 0.025). The relationship between high FADD protein expression and lymph node metastasis in head and neck cancer has been demonstrated in several studies [18, 28–29]. By contrast, Fan et al. found that the expression of *DR5*, *FADD* or both does not significantly affect the progression of HNSCC patients who have no evidence of LNM [29]. They suggested that DR5/FADD/caspase-8 signaling may have an opposite function to the previous reported in regulating cancer metastasis and may depend on the tumor stage [29]. FADD can also recruit other proteins to regulate the NF-κB and MAPK pathways, which in turn can promote proliferation and cell cycle progression.[30] In addition, the increased level of FADD transcripts was correlated with the levels of cyclin D1, which is also encoded by a gene located within the same region of 11q13. The induced NF-κB, its downstream pathway and cyclin D1 were demonstrated to be associated with poor prognosis in lung adenocarcinoma.[30]

In this study, univariate analysis indicated that high FADD expression was associated with decreased DFS and OS and that *FADD* amplification was associated with poorer OS. In the *FADD* copy neutral subgroup, high FADD expression was also an independent prognostic marker of poorer DFS. The combined effect of FADD amplification and high FADD expression was demonstrated to result in poorer DFS in patients with OSCC.

## Conclusions

FADD protein overexpression is closely associated with FADD copy number. Both FADD gene amplification and protein overexpression were associated with lymph node metastasis and perineural invasion. FADD is an important gene that is associated with lymph node metastasis and prognosis in OSCC. The combination of FADD gene amplification and protein overexpression can be successfully used as a marker to stratify patients with OSCC into risk subgroups in clinical practice.

## Supporting Information

S1 File Data file for FADD study. (SAV)

S1 Table The relationship between FADD gene copy number and protein expression. (DOCX)

S2 Table The associations between FADD protein expression and clinicopathological parameters in the FADD copy neutral subgroup of OSCC (n = 270). (DOCX)

S3 Table Univariate Cox regression model of prognostic covariates in the 270 FADD copy neutral subgroup of OSCC patients: disease-free and overall survival. (DOCX)

S4 Table Multivariate Cox regression model of prognostic covariates in the 270 patients FADD copy neutral subgroup of OSCC: disease-free and overall survival. (DOCX)

## References

[1] Santarius T, Shipley J, Brewer D, Stratton MR, Cooper CS. A census of amplified and overexpressed human cancer genes. Nat Rev Cancer. 2010;10(1):59–64. Epub 2009/12/24. nrc2771 [pii] 10.1038/nrc2771 . 20029424

[2] Huang SF, Cheng SD, Chien HT, Liao CT, Chen IH, Wang HM, et al Relationship between epidermal growth factor receptor gene copy number and protein expression in oral cavity squamous cell carcinoma. Oral Oncol. 2012;48(1):67–72. Epub 2011/08/13. S1368-8375(11)00750-0 [pii] 10.1016/j.oraloncology.2011.06.511 . 21831696

[3] Wilkerson PM, Reis-Filho JS. The 11q13-q14 amplicon: clinicopathological correlations and potential drivers. Genes Chromosomes Cancer. 2013;52(4):333–55. Epub 2012/12/12. 10.1002/gcc.22037 . 23225572

[4] Wang K, Lim HY, Shi S, Lee J, Deng S, Xie T, et al Genomic landscape of copy number aberrations enables the identification of oncogenic drivers in hepatocellular carcinoma. Hepatology. 2013;58(2):706–17. Epub 2013/03/19. 10.1002/hep.26402 . 23505090

[5] Gautschi O, Ratschiller D, Gugger M, Betticher DC, Heighway J. Cyclin D1 in non-small cell lung cancer: a key driver of malignant transformation. Lung Cancer. 2007;55(1):1–14. Epub 2006/10/31. S0169-5002(06)00530-7 [pii] 10.1016/j.lungcan.2006.09.024 . 17070615

[6] Hanken H, Grobe A, Cachovan G, Smeets R, Simon R, Sauter G, et al CCND1 amplification and cyclin D1 immunohistochemical expression in head and neck squamous cell carcinomas. Clin Oral Investig. 2014;18(1):269–76. Epub 2013/03/16. 10.1007/s00784-013-0967-6 . 23494454

[7] Reis-Filho JS, Savage K, Lambros MB, James M, Steele D, Jones RL, et al Cyclin D1 protein overexpression and CCND1 amplification in breast carcinomas: an immunohistochemical and chromogenic in situ hybridisation analysis. Mod Pathol. 2006;19(7):999–1009. Epub 2006/05/02. 3800621 [pii] 10.1038/modpathol.3800621 . 16648863

[8] Seiler R, Thalmann GN, Rotzer D, Perren A, Fleischmann A. CCND1/CyclinD1 status in metastasizing bladder cancer: a prognosticator and predictor of chemotherapeutic response. Mod Pathol. 2014;27(1):87–95. Epub 2013/07/28. modpathol2013125 [pii] 10.1038/modpathol.2013.125 . 23887292

[9] Weaver AM. Cortactin in tumor invasiveness. Cancer Lett. 2008;265(2):157–66. Epub 2008/04/15. S0304-3835(08)00158-4 [pii] 10.1016/j.canlet.2008.02.066 18406052 PMC2460566

[10] Rothschild BL, Shim AH, Ammer AG, Kelley LC, Irby KB, Head JA, et al Cortactin overexpression regulates actin-related protein 2/3 complex activity, motility, and invasion in carcinomas with chromosome 11q13 amplification. Cancer Res. 2006;66(16):8017–25. Epub 2006/08/17. 66/16/8017 [pii] 10.1158/0008-5472.CAN-05-4490 . 16912177

[11] Lavrik IN, Krammer PH. Regulation of CD95/Fas signaling at the DISC. Cell Death Differ. 2012;19(1):36–41. Epub 2011/11/15. cdd2011155 [pii] 10.1038/cdd.2011.155 22075988 PMC3252827

[12] Tourneur L, Chiocchia G. FADD: a regulator of life and death. Trends Immunol. 2010;31(7):260–9. Epub 2010/06/26. S1471-4906(10)00066-9 [pii] 10.1016/j.it.2010.05.005 . 20576468

[13] Chinnaiyan AM, O'Rourke K, Tewari M, Dixit VM. FADD, a novel death domain-containing protein, interacts with the death domain of Fas and initiates apoptosis. Cell. 1995;81(4):505–12. Epub 1995/05/19. 0092-8674(95)90071-3 [pii]. . 7538907 10.1016/0092-8674(95)90071-3

[14] Green DR, Oberst A, Dillon CP, Weinlich R, Salvesen GS. RIPK-dependent necrosis and its regulation by caspases: a mystery in five acts. Mol Cell. 2011;44(1):9–16. Epub 2011/10/11. S1097-2765(11)00714-3 [pii] 10.1016/j.molcel.2011.09.003 21981915 PMC3192321

[15] Gordy C, He YW. The crosstalk between autophagy and apoptosis: where does this lead? Protein Cell. 2012;3(1):17–27. Epub 2012/02/09. 10.1007/s13238-011-1127-x . 22314807 PMC4875212

[16] Werner MH, Wu C, Walsh CM. Emerging roles for the death adaptor FADD in death receptor avidity and cell cycle regulation. Cell Cycle. 2006;5(20):2332–8. Epub 2006/11/15. 3385 [pii]. 10.4161/cc.5.20.3385 17102623

[17] Gibcus JH, Menkema L, Mastik MF, Hermsen MA, de Bock GH, van Velthuysen ML, et al Amplicon mapping and expression profiling identify the Fas-associated death domain gene as a new driver in the 11q13.3 amplicon in laryngeal/pharyngeal cancer. Clin Cancer Res. 2007;13(21):6257–66. Epub 2007/11/03. 13/21/6257 [pii] 10.1158/1078-0432.CCR-07-1247 . 17975136

[18] Prapinjumrune C, Morita K, Kuribayashi Y, Hanabata Y, Shi Q, Nakajima Y, et al DNA amplification and expression of FADD in oral squamous cell carcinoma. J Oral Pathol Med. 2010;39(7):525–32. Epub 2009/12/31. JOP847 [pii] 10.1111/j.1600-0714.2009.00847.x . 20040024

[19] Huang SF, Cheng SD, Chuang WY, Chen IH, Liao CT, Wang HM, et al Cyclin D1 overexpression and poor clinical outcomes in Taiwanese oral cavity squamous cell carcinoma. World J Surg Oncol. 2012;10:40 10.1186/1477-7819-10-40 22336657 PMC3312822

[20] Association of Directors of A, Surgical P. Recommendations for the reporting of specimens containing oral cavity and oropharynx neoplasms. ModPathol. 2000;13(9):1038–41. 10.1038/modpathol.3880188 11007046

[21] Hsieh LL, Wang PF, Chen IH, Liao CT, Wang HM, Chen MC, et al Characteristics of mutations in the p53 gene in oral squamous cell carcinoma associated with betel quid chewing and cigarette smoking in Taiwanese. Carcinogenesis. 2001;22(9):1497–503. Epub 2001/09/05. . 11532872 10.1093/carcin/22.9.1497

[22] Bustin SA, Benes V, Garson JA, Hellemans J, Huggett J, Kubista M, et al The MIQE guidelines: minimum information for publication of quantitative real-time PCR experiments. Clin Chem. 2009;55(4):611–22. 10.1373/clinchem.2008.112797 . 19246619

[23] Sugahara K, Michikawa Y, Ishikawa K, Shoji Y, Iwakawa M, Shibahara T, et al Combination effects of distinct cores in 11q13 amplification region on cervical lymph node metastasis of oral squamous cell carcinoma. Int J Oncol. 2011;39(4):761–9. Epub 2011/06/28. 10.3892/ijo.2011.1094 . 21701773

[24] Ponder BA. Cancer genetics. Nature. 2001;411(6835):336–41. Epub 2001/05/18. [pii]. . 11357140 10.1038/35077207

[25] Muller D, Millon R, Lidereau R, Engelmann A, Bronner G, Flesch H, et al Frequent amplification of 11q13 DNA markers is associated with lymph node involvement in human head and neck squamous cell carcinomas. Eur J Cancer B Oral Oncol. 1994;30B(2):113–20. Epub 1994/01/01. . 8032300 10.1016/0964-1955(94)90062-0

[26] Cordon-Cardo C, Latres E, Drobnjak M, Oliva MR, Pollack D, Woodruff JM, et al Molecular abnormalities of mdm2 and p53 genes in adult soft tissue sarcomas. Cancer Res. 1994;54(3):794–9. Epub 1994/02/01. . 8306343

[27] Kim H, Lee HJ, Oh Y, Choi SG, Hong SH, Kim HJ, et al The DUSP26 phosphatase activator adenylate kinase 2 regulates FADD phosphorylation and cell growth. Nat Commun. 2014;5:3351 Epub 2014/02/20. ncomms4351 [pii] 10.1038/ncomms4351 24548998 PMC3948464

[28] Pattje WJ, Melchers LJ, Slagter-Menkema L, Mastik MF, Schrijvers ML, Gibcus JH, et al FADD expression is associated with regional and distant metastasis in squamous cell carcinoma of the head and neck. Histopathology. 2013;63(2):263–70. Epub 2013/06/15. 10.1111/his.12174 . 23763459

[29] Fan S, Muller S, Chen ZG, Pan L, Tighiouart M, Shin DM, et al Prognostic impact of Fas-associated death domain, a key component in death receptor signaling, is dependent on the presence of lymph node metastasis in head and neck squamous cell carcinoma. Cancer Biol Ther. 2013;14(4):365–9. Epub 2013/01/30. 23636 [pii] 10.4161/cbt.23636 23358467 PMC3667877

[30] Chen G, Bhojani MS, Heaford AC, Chang DC, Laxman B, Thomas DG, et al Phosphorylated FADD induces NF-kappaB, perturbs cell cycle, and is associated with poor outcome in lung adenocarcinomas. Proc Natl Acad Sci U S A. 2005;102(35):12507–12. Epub 2005/08/20. 0500397102 [pii] 10.1073/pnas.0500397102 16109772 PMC1194899

