# Supplementary material for: Clinical Implications of FADD Gene Amplification and Protein Overexpression in Taiwanese Oral Cavity Squamous Cell Carcinomas
Source: PLoS One. 2016 Oct 20;11(10):e0164870. doi: 10.1371/journal.pone.0164870 (PMC5072707; doi:10.1371/journal.pone.0164870)
Supplement: S1 Table — (DOCX) [file pone.0164870.s002.docx]

S1 Table. The relationship between FADD gene copy number and protein expression

| FADD expression | FADD gene copy number | | |  |
| --- | --- | --- | --- | --- |
|  | Copy neutral [N(%)] | | Amplification [N(%)] | P value* |
| Low expression | 137 (50.7) | 9 (13.0) | | < 0.001 |
| High expression | 133 (49.3) | 60 (87.0) | |  |

*χ^2^ test
