# Supplementary material for: Clinical Implications of FADD Gene Amplification and Protein Overexpression in Taiwanese Oral Cavity Squamous Cell Carcinomas
Source: PLoS One. 2016 Oct 20;11(10):e0164870. doi: 10.1371/journal.pone.0164870 (PMC5072707; doi:10.1371/journal.pone.0164870)
Supplement: S2 Table — (DOCX) [file pone.0164870.s003.docx]

S2 Table. The associations between FADD protein expression and clinicopathological parameters in the FADD copy neutral subgroup of OSCC (n=270)

|  | FADD protein expression | | |
| --- | --- | --- | --- |
|  | FADD expression-low | FADD expression-high | *P*-value |
| Age |  |  |  |
| < 50 yrs | 62 (44.3) | 78 (55.7) | **0.029** |
| > 50 yrs | 75 (57.7) | 55 (42.3) |  |
| Subsites |  |  |  |
| Bucca | 71 (55.9) | 56 (44.1) | 0.260 |
| Tongue | 38 (47.5) | 42 (52.5) |  |
| Others | 28 (44.4) | 35 (55.6) |  |
| Primary tumor status |  |  |  |
| T1/T2 | 59 (45.4) | 71 (54.6) | 0.113 |
| T3/T4 | 78 (55.7) | 62 (44.3) |  |
| Lymph node status |  |  |  |
| LNM†-/ECS‡- | 91 (57.6) | 67 (42.4) | **0.025** |
| LNM+/ECS- | 18 (38.3) | 29 (61.7) | **0.022*** |
| LNM+/ECS+ | 28 (43.1) | 37 (56.9) |  |
| Tumor differentiation |  |  |  |
| Well | 66 (58.4) | 47 (41.6) | **0.036** |
| Moderate/Poor | 71 (45.2) | 86 (54.8) |  |
| Skin invasion |  |  |  |
| Yes | 27 (65.9) | 14 (34.1) | **0.042** |
| No | 110 (48.0) | 119 (52.0) |  |
| Bone invasion |  |  |  |
| Yes | 39 (53.4) | 34 (46.6) | 0.681 |
| No | 98 (49.7) | 99 (50.3) |  |
| Perineural invasion |  |  |  |
| Yes | 30 (42.3) | 41 (57.7) | 0.100 |
| No | 107 (53.8) | 92 (46.2) |  |
| Vascular invasion |  |  |  |
| Yes | 3 (37.5) | 5 (62.5) | 0.496 |
| No | 134 (51.1) | 128 (48.9) |  |
| Lymphatic invasion |  |  |  |
| Yes | 19 (55.9) | 15 (44.1) | 0.584 |
| No | 118 (50.0) | 118 (50.0) |  |
| Invasion depth of tumor |  |  |  |
| > 10 mm | 85 (52.1) | 78 (47.9) | 0.619 |
| < 10 mm | 52 (48.6) | 55 (51.4) |  |
| Cigarette smoking |  |  |  |
| Yes | 113 (49.1) | 117 (50.9) | 0.233 |
| No | 24 (60.0) | 16 (40.0) |  |
| Alcohol drinking |  |  |  |
| Yes | 67 (47.5) | 74 (52.5) | 0.276 |
| No | 70 (54.3) | 59 (45.7) |  |
| AQ chewing |  |  |  |
| Yes | 117 (50.2) | 116 (49.8) | 0.725 |
| No | 20 (54.1) | 17 (45.9) |  |

*χ^2^ trend test; †LNM: lymph node metastasis; ^‡^ ECS: extracapsular spread
