# Supplementary material for: Clinical Implications of FADD Gene Amplification and Protein Overexpression in Taiwanese Oral Cavity Squamous Cell Carcinomas
Source: PLoS One. 2016 Oct 20;11(10):e0164870. doi: 10.1371/journal.pone.0164870 (PMC5072707; doi:10.1371/journal.pone.0164870)
Supplement: S3 Table — (DOCX) [file pone.0164870.s004.docx]

S3 Table. Univariate Cox regression model of prognostic covariates in the 270 FADD copy neutral subgroup of OSCC patients: disease-free and overall survival

| Characteristics | DFS HR (95% CI) | *P*-value | OS HR (95% CI) | *P*-value |
| --- | --- | --- | --- | --- |
| Age |  |  |  |  |
| < 50 yrs | 1 |  | 1 |  |
| > 50 yrs | 0.864 (0.602-1.238) | 0.425 | 1.148 (0.827-1.592) | 0.410 |
| Primary tumor status |  |  |  |  |
| T1/T2 | 1 |  | 1 |  |
| T3/T4 | 1.036 (0.723-1.484) | 0.847 | 1.753 (1.255-2.448) | **0.001** |
| Lymph node status |  |  |  |  |
| LNM†-/ECS‡- | 1 |  | 1 |  |
| LNM+/ECS- | 1.636 (1.000-2.678) | 0.050 | 1.825 (1.175-2.836) | **0.007** |
| LNM+/ECS+ | 2.908 (1.937-4.365) | **<0.001** | 2.812 (1.937-4.081) | **<0.001** |
| Tumor differentiation |  |  |  |  |
| Well | 1 |  | 1 |  |
| Moderate/Poor | 1.038 (0.723-1.490) | 0.840 | 1.256 (0.899-1.754) | 0.182 |
| FADD expression |  |  |  |  |
| Low expression | 1 |  | 1 |  |
| High expression | 1.690 (1.173-2.435) | **0.005** | 1.283 (0.924-1.780) | 0.136 |

†LNM: lymph node metastasis; ^‡^ ECS: extracapsular spread
