# Supplementary material for: Clinical Implications of FADD Gene Amplification and Protein Overexpression in Taiwanese Oral Cavity Squamous Cell Carcinomas
Source: PLoS One. 2016 Oct 20;11(10):e0164870. doi: 10.1371/journal.pone.0164870 (PMC5072707; doi:10.1371/journal.pone.0164870)
Supplement: S4 Table — (DOCX) [file pone.0164870.s005.docx]

S4 Table. Multivariate Cox regression model of prognostic covariates in the 270 patients FADD copy neutral subgroup of OSCC: disease-free and overall survival

| Characteristics | DFS HR (95% CI) | *P*-value | OS HR (95% CI) | *P*-value |
| --- | --- | --- | --- | --- |
| Age |  |  |  |  |
| < 50 yrs | 1 |  | 1 |  |
| > 50 yrs | 1.043 (0.719-1.514) | 0.823 | 1.235 (0.883-1.725) | 0.217 |
| Primary tumor status |  |  |  |  |
| T1/T2 | 1 |  | 1 |  |
| T3/T4 | 0.945 (0.654-1.365) | 0.762 | 1.607 (1.143-2.259) | **0.006** |
| Lymph node status |  |  |  |  |
| LNM†-/ECS‡- | 1 |  | 1 |  |
| LNM+/ECS- | 1.572 (0.947-2.609) | 0.080 | 1.780 (1.132-2.799) | **0.012** |
| LNM+/ECS+ | 3.089 (1.987-4.803) | **<0.001** | 2.637 (1.773-3.921) | **<0.001** |
| Tumor differentiation |  |  |  |  |
| Well | 1 |  | 1 |  |
| Moderate/Poor | 0.741 (0.505-1.089) | 0.127 | 0.972 (0.684-1.381) | 0.875 |
| FADD expression |  |  |  |  |
| Low expression | 1 |  | 1 |  |
| High expression | 1.575 (1.078-2.300) | **0.019** | 1.255 (0.894-1.762) | 0.190 |

†LNM: lymph node metastasis; ^‡^ ECS: extracapsular spread
